# Identification of vulnerable non-culprit lesions by coronary computed tomography angiography in patients with chronic coronary syndrome and diabetes mellitus

**DOI:** 10.3389/fcvm.2023.1143119

**Published:** 2023-03-23

**Authors:** Jia Zhao, Hong Zhang, Chang Liu, Ying Zhang, Cun Xie, Minghui Wang, Chengjian Wang, Shuo Wang, Yuanyuan Xue, Shuo Liang, Yufan Gao, Hongliang Cong, Chunjie Li, Jia Zhou

**Affiliations:** ^1^ Clinical School of Thoracic, Tianjin Medical University, Tianjin, China; ^2^ Department of Cardiology, Tianjin Chest Hospital, Tianjin, China; ^3^ Department of Radiology, Tianjin Chest Hospital, Tianjin, China

**Keywords:** chronic coronary syndrome, non-culprit lesion, coronary computed tomography angiography, adverse plaque characteristic, major adverse cardiovascular event, diabetes mellitus

## Abstract

**Background:**

Among patients with diabetes mellitus (DM) and chronic coronary syndrome (CCS), non-culprit lesions (NCLs) are responsible for a substantial number of future major adverse cardiovascular events (MACEs). Thus, we aimed to establish the natural history relationship between adverse plaque characteristics (APCs) of NCLs non-invasively identified by coronary computed tomography angiography (CCTA) and subsequent MACEs in these patients.

**Methods:**

Between January 2016 and January 2019, 523 patients with DM and CCS were included in the present study after CCTA and successful percutaneous coronary intervention (PCI). All patients were followed up for MACEs (the composite of cardiac death, myocardial infarction, and unplanned coronary revascularization) until January 2022, and the independent clinical event committee classified MACEs as indeterminate, culprit lesion (CL), and NCL-related. The primary outcome was MACEs arising from untreated NCLs during the follow-up. The association between plaque characteristics detected by CCTA and primary outcomes was determined by Marginal Cox proportional hazard regression.

**Results:**

Overall, 1,248 NCLs of the 523 patients were analyzed and followed up for a median of 47 months. The cumulative rates of indeterminate, CL, and NCL-related MACEs were 2.3%, 14.5%, and 20.5%, respectively. On multivariate analysis, NCLs associated with recurrent MACEs were more likely to be characterized by a plaque burden >70% [hazard ratio (HR), 4.35, 95% confidence interval (CI): 2.92–6.44], a low-density non-calcified plaque (LDNCP) volume >30 mm^3^ (HR: 3.40, 95% CI: 2.07–5.56), a minimal luminal area (MLA) <4 mm^2^ (HR: 2.30, 95% CI: 1.57–3.36), or a combination of three APCs (HR: 13.69, 95% CI: 9.34–20.12, *p* < 0.0001) than those not associated with recurrent MACEs. Sensitivity analysis regarding all indeterminate MACEs as NCL-related ones demonstrated similar results.

**Conclusions:**

In DM patients who presented with CCS and underwent PCI, half of the MACEs occurring during the follow-up were attributable to recurrence at the site of NCLs. NCLs responsible for unanticipated MACEs were frequently characterized by a large plaque burden and LDNCP volume, a small MLA, or a combination of these APCs, as determined by CCTA.

## Background

Despite dramatic advancements in pharmacotherapy and stent technology, recurrent major adverse cardiovascular events (MACEs) after percutaneous coronary intervention (PCI) remain common and create management challenges in patients with diabetes mellitus (DM) (1, 2). Compared to those without DM, the increased risk of patients with DM and acute or chronic coronary syndrome (ACS or CCS) should be attributable to the larger burden of coronary plaque and more unremitting and rapidly progressive atherosclerosis progression (3), which would cause more MACEs related to nonculprit lesions (NCLs) deferred for PCI (4–10). Recent intracoronary imaging-based studies revealed that NCLs with specific adverse plaque characteristics (APCs) could lead to unanticipated MACEs in their natural history (4, 5, 9, 11–14). However, these invasive imaging modalities are difficult to generalize in clinical practice, especially for comprehensive imaging of the whole coronaries. In addition, the number of patients with DM and CCS for whom the selection of an imaging-based management strategy was important but challenging was relatively low in these studies (4–9, 14, 15). In DM patients who presented with CCS and underwent PCI, half of the MACEs occurring during the follow-up were attributable to recurrence at the site of NCLs.

Non-invasive analysis of APCs based on coronary computed tomography angiography (CCTA) has been validated against intravascular ultrasonography (IVUS) (16), optical coherence tomography (OCT) (17), and near-infrared spectroscopy (NIRS) (18), and it showed important value in the prediction of future MACEs (6, 19–26). However, these CCTA-based studies investigating the association between APCs and prognosis were susceptible to clinical intervention, such as medication and PCI, leading to the uncertainty of lesions responsible for unanticipated MACEs (6, 19–25). Thus, the purpose of this study is to evaluate whether relating clinical factors and CCTA-derived baseline APCs to subsequent MACEs can provide early and robust identification of NCLs, which would lead to unanticipated MACEs in their long-term natural history among patients with DM and CCS after successful PCI for culprit lesions (CLs).

## Materials and Methods

### Study population

**Figure 1:**
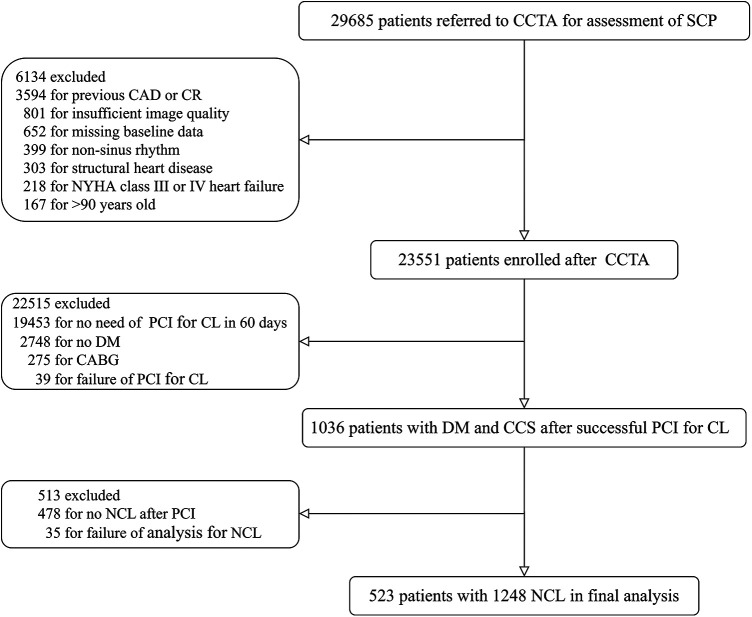
Consort diagram.

The CCTA Improves Clinical Management of Stable Chest Pain (CICM-SCP) registry is an ongoing registry of patients referred to CCTA for the assessment of stable chest pain suspected of CCS (NCT04691037). Details about the registry, including inclusion and exclusion criteria, have been described previously (27, 28). As illustrated in Figure 1, between January 2016 and January 2019, 23,551 patients were enrolled after CCTA in the registry after excluding patients with previous CAD, insufficient image quality, missing baseline data, non-sinus rhythm, structural heart disease, heart failure, or >90 years old. Among patients undergoing coronary angiography based on the results of CCTA, 1,036 patients with DM and CCS completed successful PCI for CLs. Patients were considered as suffering from DM if one of the following criteria was met: treatment with insulin or hypoglycemic medications, fasting blood glucose ≥7.0 mmol/L, a 2 h plasma glucose level in their oral glucose tolerance test ≥11.1 mmol/L, or a glycated hemoglobin value ≥6.5%. Other baseline clinical data were defined and collected as described previously (27, 28) and detailed in the Supplementary Material. After excluding 478 patients with no NCL left and 35 for failure imaging analysis of NCL, 523 patients with 1,248 NCLs were included in the present study. This observational study was approved by the Local Ethics Committees (Tianjin Chest Hospital, 2017-KY-004).

### Imaging protocols and analyses

Image scanning, data collection, and result interpretation of CCTA and invasive coronary angiography (ICA) were conducted according to the established guideline (29) and local protocols as previously described (27, 28) and detailed in the Supplementary Material. CLs were identified based on a combination of angiographic appearance (both CCTA and ICA), results of other non-invasive or invasive functional testing, electrocardiograph findings, electrocardiographic left ventricular wall motion abnormalities, or lesion morphology detected by intracoronary imaging and treated by PCI according to the recommendations in current guidelines (30, 31) and clinical practice at the local institution. According to the definitions from previous studies (6, 16, 19–21, 32) and established guidelines (29), qualitative and quantitative analyses of APCs, including area stenosis >50%, minimal luminal area (MLA) <4 mm^2^, plaque burden >70%, low-density non-calcified plaque (LDNCP) volume >30 mm^3^, positive remodeling, spotty calcification, and napkin-ring sign (NRS), are detailed in the Supplementary Material.

### Follow-up and study endpoint

A MACE is defined as a composite of cardiac death, non-fatal myocardial infarction, and unplanned coronary revascularization for recurrent ACS with plaque progression. Details about the definition of the study endpoint and follow-up information collection have been described previously (27, 28) and are presented in the Supplementary Material. At the time of MACE, based on available imaging of follow-up ICA and other clinical data such as hospital records and information provided by physicians, patients, and relatives, an independent clinical event committee who was blinded to other data classified a MACE as attributable to a culprit lesion (CL) originally treated by PCI, or, to a medically treated NCL. When data were not sufficient to classify a MACE as either CL-related or NCL-related, the MACE was classified as indeterminate. Then, the NCL was coregistered to the baseline CCTA precursor lesion by comparison of coronary segment coding and using distance from ostia and coronary vessel branch points as fiduciary landmarks. The primary endpoint was a MACE arising from an untreated NCL. If the patients were lost to follow-up or died from non-cardiac disease, they would be censored. For patients suffering from multiple MACEs, only the first MACE of each classification (CL-related, NCL-related, or indeterminate) was analyzed.

### Statistical analysis

All statistical analyses were performed using R (version 3.2.4; R Foundation for Statistical Computing, Vienna, Austria) and MedCalc (version 15.2.2; MedCalc Software, Mariakerke, Belgium). Two-tailed *p* < 0.05 was considered statistically significant. Differences in continuous data were compared using Student's *t*-test or the Mann–Whitney *U*-test as appropriate. Categorical variables were compared using the *χ*^2^ test or Fisher exact test as appropriate.

For patient-level analyses, we constructed Kaplan–Meier curves for cumulative event-free survival estimates from the first MACE of each classification and all. For lesion-level analyses, we used a generalized estimating equation to adjust for intrasubject variability among lesions from the same patient. Marginal Cox proportional hazard regression was used to assess the association of predictors to the time to the first NCL-related MACE or censoring. Given the number of study endpoints available, baseline variables that were used in similar studies, were clinically relevant, or showed a univariate relationship with MACEs (*p* < 0.10) were parsimoniously included in the multivariate model. MLA < 4 mm^2^, plaque burden >70%, and LDNCP volume >30 mm^3^ were prespecified for use in the multivariate regression model since they have been used frequently in previous studies (4–6, 29, 32). The results were presented as hazard ratios (HRs) and 95% confidence intervals (CIs). We also constructed forest plots to intuitively illustrate HRs of different combinations of APCs with 95% CIs and *p*-values due to the recommendation from current guidelines, which defined a lesion with two or more APCs as a vulnerable one (29). For sensitivity analysis, we classified all indeterminate MACEs as NCL-related MACEs (the NCL with the highest degree of area stenosis at baseline was considered the lesion responsible for recurrent MACE) and investigated the association between APC and study endpoint. The predictive performances of these parameters were evaluated using sensitivity, specificity, positive or negative predictive value (PPV or NPV), and C statistic with 95% CI.

## Results

Table 1 shows the baseline clinical and procedural characteristics of the study cohort. The mean age was 64.6 years, 67% were men, and 76% had multivessel diseases. Hyperlipidemia, the number of diseased vessels, and insulin therapy were significantly higher in patients with MACEs related to NCLs, (*p* = 0.0098, *p* < 0.0001, and *p* = 0.0127, respectively). The proportions of other baseline investigations did not differ significantly between the two groups.

**Table 1:** Clinical and procedural characteristics of patients.

Characteristic	Total	MACE related to NCL		*p*
	*N* = 523	Yes (*N* = 107)	No (*N* = 416)	
Age^a^	64.6 ± 11.2	65.1 ± 12.7	64.5 ± 10.9	0.6241
Male	451 (67)	78 (73)	273 (66)	0.1893
BMI^b^	29.3 ± 7.5	29.8 ± 9.1	29.2 ± 7.8	0.4937
Hypertension	387 (74)	84 (79)	303 (73)	0.2853
Hyperlipidemia	417 (61)	77 (72)	240 (58)	0.0098
Smoking	231 (44)	54 (50)	177 (43)	0.1732
Family history	271 (52)	63 (59)	208 (50)	0.1258
Cerebrovascular disease	94 (18)	24 (22)	70 (17)	0.2282
PVD	67 (13)	14 (13)	53 (13)	0.9464
Renal insufficiency	32 (6)	11 (10)	21 (5)	0.0738
Other baseline investigations	396 (76)	76 (71)	320 (74)	0.2535
Functional testing	277 (53)	54 (50)	223 (54)	0.6373
Non-invasive	151 (29)	36 (34)	115 (28)	0.2705
Invasive	46 (9)	5 (5)	41 (10)	0.1239
Intracoronary imaging	45 (9)	13 (12)	32 (8)	0.2030
No. of diseased vessels^c^				<0.0001
One	125 (24)	8 (7)	117 (28)	
Two	215 (41)	51 (48)	164 (39)	
Three	183 (35)	48 (45)	135 (33)	
Syntax score^d^	15.5 ± 5.4	16.5 ± 8.1	15.2 ± 6.7	0.0876
No. of implanted stents^d^	1.9 ± 1.2	2.1 ± 1.8	1.8 ± 1.4	0.0638
Medicine treatment at baseline				
Antiplatelet therapy	335 (64)	69 (64)	266 (64)	0.9933
Statin	302 (58)	66 (62)	236 (57)	0.4151
Insulin therapy	314 (60)	76 (71)	238 (57)	0.0127
Beta-blocker	248 (49)	54 (50)	194 (46)	0.5488
ACEI/ARB	367 (70)	72 (67)	295 (71)	0.5404
Calcium channel blocker	363 (69)	81 (76)	282 (68)	0.1425

PVD, peripheral vascular disease; ACEI, angiotensin-converting enzyme inhibitor; ARB, angiotensin II receptor blocker.

Values are presented as *n* (%) unless stated otherwise.

^a^ Years, mean ± standard deviation.

^b^ kg/m^2^, mean ± standard deviation.

^c^ This category refers to any lesion with stenosis ≥30% of vessel diameter.

^d^ Mean ± standard deviation.

**Figure 2:**
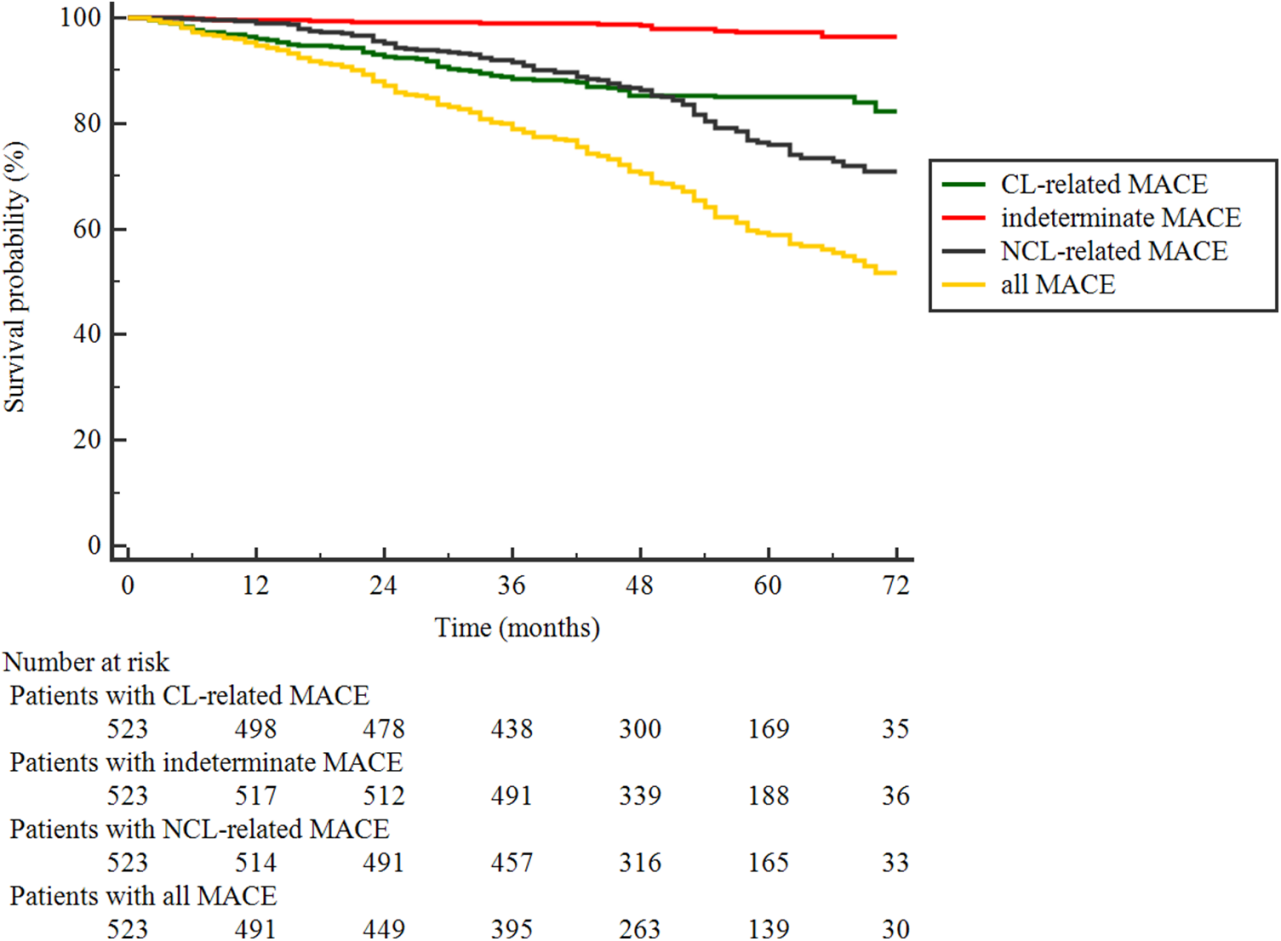
Kaplan–Meier curves of patients surviving event-free from the first MACE of each classification and all.

Patients were followed up for a median of 47 (interquartile range: 32–61) months, and 28 (5.4%) patients were lost. During the follow-up, 195 MACEs occurred in 149 of 523 (28.5%) patients. As shown in Table 2, 76 (14.5%) MACEs were recurrent diseases at originally treated CLs, 107 (20.5%) were related to NCLs, and the origin of 12 (2.3%) was indeterminate. Among the 107 NCL-related MACEs, most (16.6%, 87/107) were unplanned revascularization for unstable or progressive angina; only three patients (0.6%) died from cardiac causes, and 25 patients (4.8%) suffered from a non-fatal myocardial infarction. The Kaplan–Meier curves in Figure 2 illustrated that the discrepancy of cumulative rates between the CL and NCL-related MACEs appeared to be mainly attributed to the more frequent occurrences of NCL-related MACEs in the later stage of follow-up.

**Table 2:** Study endpoints related to CL, NCL, or indeterminate MACEs.

	Events related to CL	Events related to NCL	Indeterminate events
Death from cardiac causes	2 (0.4)	3 (0.6)	7 (1.3)
Non-fatal MI	21 (4.0)	25 (4.8)	4 (0.8)
Unplanned revascularization	53 (10.1)	79 (15.1)	1 (0.2)
Total	76 (14.5)	107 (20.5)	12 (2.3)

CL, culprit lesion; NCL, non-culprit lesion; MI, myocardial infarction.

Values are presented as *n* (%) unless stated otherwise.

**Figure 3:**
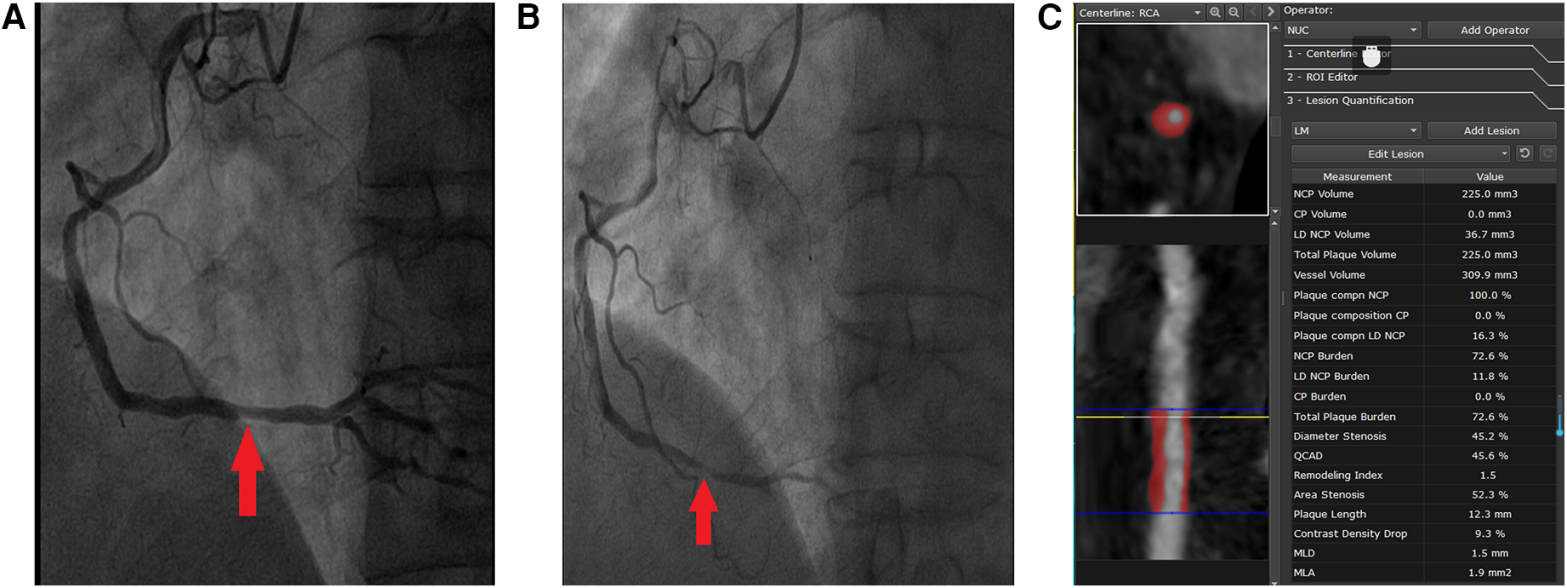
Representative case of a MACE attributed to the progression of an NCL that was not treated by PCI at baseline. A 51-year-old man presented with CCS, DM (insulin therapy), hypertension, smoking, and three-vessel disease (syntax score: 19). Successful PCI of CL that had caused CCS in the proximal segment of left anterior descending was done. One angiographically mild lesion was identified in the distal segment of the right coronary artery (arrow in **A**), and PCI treatment of this lesion was deferred. However, the patient re-presented with MI 3 years later. ICA showed a new severe stenosis with pronounced lesion progression and thrombus at the site of the originally untreated NCL in the distal segment of the right coronary artery (arrow in **B**). The imaging analysis of baseline CCTA by the core laboratory showed that this NCL was a completely non-calcified plaque with positive remodeling (**C**). The MLA was 1.9 mm^2^, plaque burden was 72.6%, and the volume of LDNCP was 36.7 mm^3^.

Table 3 shows the CCTA-measured plaque characteristics of NCLs with and without MACEs. At baseline, 1,248 NCLs were identified with a mean area stenosis of 49.79%, a mean MLA of 4.16 mm^2^, and a mean plaque burden of 59.57%. The NCLs with MACEs had higher area stenosis, plaque burden, and remodeling index and lower MLA, resulting in area stenosis >50%, LDNCP volume >30 mm^3^, plaque burden >70%, positive remodeling, and MLA < 4 mm^2^. In addition, more potty calcification (39% vs. 24%, *p* < 0.0001) and NRS (22% vs. 8%, *p* < 0.0001) were found in NCLs with MACEs. Figure 3 shows a representative example of a MACE arising from an NCL.

**Table 3:** CCTA-measured plaque characteristics of NCLs.

Characteristic	Total	MACE related to NCL		*p*
	*N* = 1,248	Yes (*N* = 107)	No (*N* = 1,141)	
**Location**				
LMCA	84 (7)	9 (8)	75 (6)	0.6004
Proximal segment of LAD	288 (23)	35 (33)	253 (22)	0.0186
vProximal segment of LCX	239 (19)	13 (12)	226 (20)	0.0724
Proximal segment of RCA	154 (12)	20 (19)	134 (12)	0.0529
**Quantitative parameters**				
Area stenosis, %	49.79 ± 13.68	61.21 ± 18.24	48.72 ± 11.72	<0.0001
MLA, mm^2^	4.16 ± 2.26	2.94 ± 1.53	4.28 ± 1.87	<0.0001
Plaque burden, %	59.57 ± 12.83	72.62 ± 16.49	58.35 ± 11.24	<0.0001
NCP burden, %	39.96 ± 27.61	61.73 ± 40.33	37.92 ± 22.59	<0.0001
LDNCP burden, %	3.20 ± 3.35	10.86 ± 6.92	2.48 ± 2.65	<0.0001
Remodeling index	1.18 ± 0.49	1.44 ± 0.71	1.16 ± 0.38	<0.0001
Lesion length, mm	9.58 ± 6.97	11.25 ± 7.41	10.03 ± 6.35	0.0615
**Qualitative parameters**				
Area stenosis >50%	330 (26)	48 (45)	282 (25)	<0.0001
MLA < 4 mm^2^	560 (45)	76 (71)	484 (42)	<0.0001
Plaque burden >70%	340 (27)	72 (67)	268 (23)	<0.0001
LDNCP volume >30 mm^3^	525 (42)	80 (75)	445 (39)	<0.0001
Positive remodeling	423 (34)	53 (50)	370 (32)	<0.0001
Spotty calcification	320 (26)	42 (39)	278 (24)	<0.0001
NRS	112 (9)	24 (22)	88 (8)	<0.0001

LMCA, left main coronary artery; LAD, left anterior descending; LCX, left circumflex; RCA, right coronary artery; CCTA, coronary computed tomographic angiography; MACE, major adverse cardiovascular event; NCL, non-culprit lesion; MLA, minimum lumen area; NCP, non-calcified plaque; LDNCP, low-density non-calcified plaque; NRS, napkin-ring sign.

Values are presented as *n* (%) or mean ± standard deviation.

**Figure 4:**
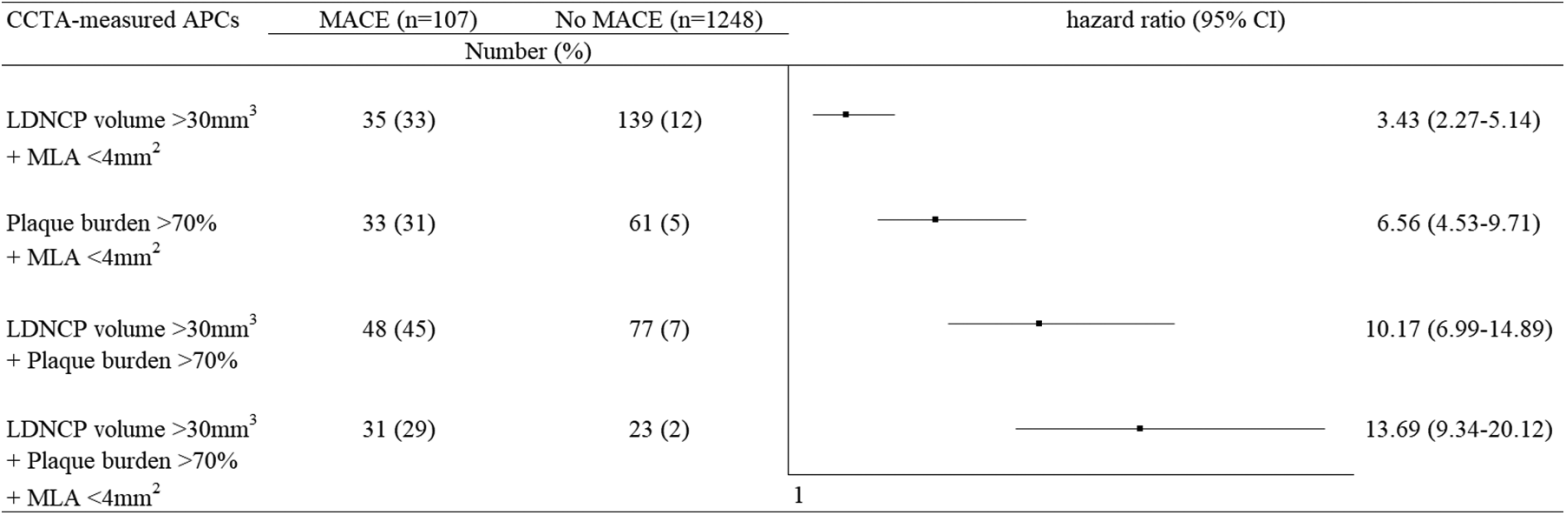
Association between the study endpoints and different combinations of APCs of NCL.

Table 4 shows the univariate and multivariate analysis results for predictors of NCL-related MACEs. The assumption of proportionality was checked using Schoenfeld residual tests, and all models satisfied the assumption of proportional hazards. Univariate Cox models revealed that three diseased vessels, insulin therapy, the proximal segment of LAD, area stenosis >50%, MLA < 4 mm^2^, plaque burden >70%, LDNCP volume >30 mm^3^, positive remodeling, spotty calcification, and NRS were associated with the occurrence of NCL-related MACEs. However, after adjusting for covariables, only three diseased vessels (HR: 2.09, 95% CI: 1.16–3.39, *p* < 0.0001) at the patient level and MLA < 4 mm^2^ (HR: 2.30, 95% CI: 1.57–3.36, *p* < 0.0001), plaque burden >70% (HR: 4.35, 95% CI: 2.92–6.44, *p* < 0.0001), and LDNCP volume >30 mm^3^ (HR: 3.40, 95% CI: 2.07–5.56, *p* < 0.0001) at the lesion level were independent predictors of subsequent NCL-related MACEs. As illustrated in Figure 4, of the 107 NCLs that resulted in MACEs, 31 (29%) simultaneously had plaque burden >70%, LDNCP volume >30 mm^3^, and MLA < 4 mm^2^. More importantly, MACEs arose more frequently from NCLs that had a plaque burden >70% with MLA < 4 mm^2^ (HR: 6.56, 95% CI: 4.53–9.71, *p* < 0.0001), LDNCP volume >30 mm^3^ (HR: 10.17, 95% CI: 6.99–14.89, *p* < 0.0001), or both (HR: 13.69, 95% CI: 9.34–20.12, *p* < 0.0001). The sensitivity analysis regarding all indeterminate MACEs as NCL-related MACEs demonstrated similar results and is detailed in Supplementary Figure S1.

**Table 4:** Univariate and multivariate analyses for predictors of NCL-related MACE.

	Univariate		Multivariate	
	HR (95% CI)	*p*	HR (95% CI)	*p*
**Patient-level predictors** ^a^				
Hyperlipidemia	1.52 (0.91–2.38)	0.0923	1.08 (0.69–1.72)	0.7126
No. of diseased vessels^b^				
One	reference	–	reference	–
Two	1.12 (0.74–1.75)	0.3275	0.96 (0.52–1.58)	0.9351
Three	2.51 (1.61–3.57)	<0.0001	2.09 (1.16–3.39)	<0.0001
Insulin therapy	1.73 (1.05–2.81)	0.0382	1.14 (0.81–1.78)	0.3741
**Lesion-level predictors** ^c^				
Proximal segment of LAD	1.71 (1.11–2.62)	0.0064	1.08 (0.65–1.82)	0.6002
Area stenosis >50%	2.26 (1.55–3.30)	<0.0001	1.58 (0.82–2.76)	0.1989
MLA < 4 mm^2^	3.05 (2.02–4.61)	<0.0001	2.30 (1.57–3.36)	<0.0001
Plaque burden >70%	6.35 (4.24–9.51)	<0.0001	4.35 (2.92–6.44)	<0.0001
LDNCP volume >30 mm^3^	3.93 (2.62–6.14)	<0.0001	3.40 (2.07–5.56)	<0.0001
Positive remodeling	1.81 (1.24–2.63)	0.0021	1.32 (0.71–2.23)	0.5281
Spotty calcification	1.70 (1.15–2.51)	0.0081	1.06 (0.54–2.00)	0.8053
NRS	3.09 (1.97–4.85)	<0.0001	1.72 (0.89–3.34)	0.0805

MACE, major adverse cardiovascular event; NCL, non-culprit lesion; MLA, minimum lumen area; NCP, non-calcified plaque; LDNCP, low-density non-calcified plaque; NRS, napkin-ring sign; HR, hazard ratio; LAD, left anterior descending; CI, confidence interval.

^a^ The final variables entered into the patient-level multivariate regression model were age, sex, hypertension, hyperlipidemia, smoking, family history, cerebrovascular disease, PVD, renal insufficiency, number of diseased vessels, syntax score, number of implanted stents, and medicine treatment at baseline.

^b^ This category refers to any lesion with stenosis ≥30% of vessel diameter.

^c^ The final variables entered into the lesion-level multivariate regression model were the proximal segment of LAD, area stenosis >50%, MLA < 4 mm^2^, plaque burden >70%, LDNCP volume >30 mm^3^, positive remodeling, spotty calcification, and NRS as well as the significant patient-level predictors.

Table 5 reports the performance measures of diagnostic accuracy to predict MACEs for different APCs of NCLs. Plaque burden >70% was the strongest lesion-level parameter to predict NCL-related MACEs, with a C statistic of 0.72, a specificity of 76.51%, and a sensitivity of 67.29%. All three single parameters offered low specificities and positive predictive values, whereas their combinations were associated with higher specificities and positive predictive values at the expense of a decrease in sensitivity.

**Table 5:** Diagnostic metrics of different APCs of NCLs to predict MACEs.

	Sensitivity (%)	Specificity (%)	Negative predictive value (%)	Positive predictive value (%)	C statistic
Plaque burden >70%	67.29 (57.55–76.05)	76.51 (73.49–78.94)	96.15 (94.68–97.30)	21.18 (16.95–25.91)	0.72 (0.69–0.74)
LDNCP volume >30 mm^3^	74.77 (65.45–82.67)	61.00 (58.10–63.84)	96.27 (94.61–97.52)	15.24 (12.27–18.60)	0.68 (0.65–0.70)
MLA < 4 mm^2^	71.03 (61.46–79.39)	57.58 (54.65–60.47)	95.49 (93.67–96.92)	13.57 (10.84–16.69)	0.64 (0.62–0.67)
Plaque burden >70% + MLA < 4 mm^2^	30.84 (22.27–40.50)	94.65 (93.19–95.89)	93.59 (92.02–94.93)	35.11 (25.54–45.64)	0.63 (0.60–0.65)
Plaque burden >70% + LDNCP volume >30 mm^3^	44.86 (35.23–54.78)	93.25 (91.64–94.64)	94.75 (93.28–95.98)	38.40 (29.84–47.52)	0.69 (0.66–0.72)
LDNCP volume >30 mm^3 ^+ MLA < 4 mm^2^	32.71 (23.95–42.45)	87.82 (85.78–89.66)	93.30 (91.63–94.72)	20.11 (14.43–26.85)	0.60 (0.57–0.63)
Plaque burden >70% + MLA < 4 mm^2^					
+LDNCP volume >30 mm^3^	28.97 (20.61–38.54)	97.98 (96.99–98.72)	93.63 (92.10–94.95)	57.41 (43.21–70.77)	0.63 (0.61–0.66)

APC, adverse plaque characteristic; MACE, major adverse cardiovascular event; NCL, non-culprit lesion; MLA, minimum lumen area; LDNCP, low-density non-calcified plaque; CI, confidence interval.

Values are presented as numbers (95% CI).

## Discussion

The present study investigated the prognostic value of qualitative or quantitative APCs from CCTA for the occurrence of NCL-related MACEs in patients with DM and CCS. The findings of this real-world study, in which patients with DM underwent three-vessel imaging before successful PCI for CLs, showed that half of the MACEs during the 4-year follow-up arose from medically treated NCLs and that the NCLs most likely to cause a subsequent MACE had a large plaque burden, high lipid content, and low lumen area, as detected by CCTA non-invasively. The association of plaque burden >70% with NCL-related MACEs was strongest among the three APCs, leading to the best performance in predicting NCL-related MACEs.

Current evidence demonstrated a similar or even higher rate of recurrent MACEs arising from the previously untreated NCLs during the follow-up compared with CLs after successful PCI (4, 7–9), which was also supported by the present study. Thus, identification of NCLs at risk by imaging testing may improve risk stratification and management, which is particularly critical for DM patients with complex CAD, given the generalized distribution, increased complexity, and rapid progression of coronary atherosclerosis (3). In prior studies, IVUS-derived great plaque burden and small MLA (4, 5, 9), OCT-derived thin-cap fibroatheromas (TCFA) (11, 14) and calcified nodule (13), and NIRS-derived lipid-rich necrotic core (9, 12) have been identified, but these approaches have not been widely adopted in daily practice due to their invasive nature, relatively low positive predictive value, and unclear cost-effectiveness (33).

Although the spatial resolution is still limited, advancements in CCTA have allowed for the quantification of coronary atherosclerotic characteristics throughout the entire coronary, with high diagnostic performance compared with invasive reference standards (16–18). Moreover, recent studies showed that the additive value of CCTA-defined APCs in predicting future MACEs (6, 19–24) is consistent with the results of univariate regression analysis in the present study. Unfortunately, the increased risk associated with positive remodeling, spotty calcification, or NRS was not observed after adjustment for patient- and lesion-level predictors. Similarly, the EMERALD study, which investigated the APCs that would eventually be responsible for an acute event and compared the information gain of all potential APCs in a retrospective CCTA-based cohort, demonstrated that diameter stenosis showed a higher rank than remodeling index, NRS, or spotty calcification (34).

The following should emerge as particularly strong candidates for mechanistic explanations for the paradox, the first two of which were also the fundamental uniqueness of this study. First, most invasive imaging modality-based studies only included ACS patients with a low proportion of DM patients and short- to medium-term follow-up (5, 9, 11–13). It should be noted that the long-term follow-up results of the ATHEROREMO-IVUS study, which included both CCS and ACS patients, demonstrated that the single isolated features of plaque composition as derived by radiofrequency-IVUS could not predict NCL-related MACEs on their own (4). Second, previous CCTA-based studies were susceptible to the absent determination of lesions accounting for MACEs (19–22). In other words, they were not natural history studies because of the possible PCI between CCTA and MACE at the lesion, usually with high-grade stenosis. Thus, whether the subsequent MACE was attributed to CLs originally treated by PCI or to medically treated NCLs remained undiscovered. Halon et al. conducted a natural history study in asymptomatic patients with DM, leading to a low rate of MACEs during the long-term follow-up (35). Rather than the HR of an individual APC, another study using fractional flow reserve to determine NCL only provided HR based on the number of APCs, and the proportion of DM patients was also low (6). Third, due to limitations with respect to spatial resolution, controversy exists about the ability of CCTA to correctly identify some parameters of vulnerable plaque, such as quantification of spotty calcification, fibrous cap (36, 37), and remodeling index in a small diameter vessel (29). The recent COMBINE FFR-OCT study found that patients with OCT-derived TCFAs had a 5-fold higher rate of MACEs despite the absence of ischemia detected by fractional flow reserve (14). Fourth, whereas plaque burden has been shown as a consistent and robust predictor of a MACE (19, 22, 23, 33), few studies have yet demonstrated that other APCs, especially positive remodeling by themselves, independently predicted MACEs after adjustment for plaque burden and other potential confounders.

With the validation of previous findings showing that local APCs might not identify vulnerable lesions efficiently for the low rate of MACEs in terms of a large number of lesions with APCs (4–6, 9, 11–14, 19–23, 33), the present study demonstrated three independent predictors with high NPVs but low PPVs for future MACEs. Furthermore, PPVs increased moderately at the expense of dramatically decrease in sensitivities when we tried to improve PPVs by a combination of the individual APCs. Similarly limited to the low rate of NCL-related MACEs, the PROSPECT-ABSORB study comparing PCI + medical treatment with medical treatment alone for NCL with APCs was not powered for clinical events (38). On the contrary, various systemic therapies have been proposed to reduce plaque vulnerability, e.g., novel lipid-lowering and glucose-lowering, anti-inflammatory, and intensive antiplatelet therapies (33, 39, 40). Coronary atherosclerosis is influenced by an array of systemic factors *per se*, from plaque characteristics to the burden of atherosclerotic disease, its metabolic activity, and the body's response to atherosclerosis disruption (33, 41, 42). Moreover, the ISCHEMIA trial did not find evidence that an initial invasive strategy, as compared with an initial conservative strategy, reduced the risk of MACEs among patients with CCS, even in the DM subgroup (HR: 0.92, 95% CI: 0.74–1.15) (43). As a result, the non-invasive identification of vulnerable NCL with APCs by CCTA may constitute an optimal risk stratification tool to guide post-PCI management of patients with DM and CCS, including lifestyle modifications and intensive pharmacologic treatments (15). On the contrary, among high-risk patients (69.8% had a multivessel disease and 38.7% had diabetes) who had undergone PCI, a follow-up strategy of routine functional testing, as compared with standard care alone, did not improve clinical outcomes (44).

Several limitations merit consideration. First, this was a subgroup analysis of an observational and natural history registry. The influence of potential bias could not be completely excluded. Indications for testing and post-testing management relied on the decision-making of local physicians in a non-randomized fashion. The identification of CL and the decision to perform or defer PCI were at the discretion of the operators (7). Second, as the current study included patients with relatively low risk, which was supported by a low SYNTAX score, the NCL-related MACEs were mainly driven by unplanned coronary revascularization rather than cardiac death or myocardial infarction, like previous studies described (4–6, 9, 11–13, 19–22, 33). However, all MACEs were adjudicated by an independent event adjudication committee, and most were associated with objective evidence of disease progression. Moreover, the sensitivity analysis regarding all indeterminate MACEs as NCL-related MACEs demonstrated similar results. Third, the relatively low number of NCLs in the present study should be mainly attributed to the risk profile of the population and the limitations of CCTA in spatial resolution to identify NCL in vessels with small diameters (5). Fourth, our results might not be applicable to patients with ACS or without DM, who could have a different level of CAD burden, systemic inflammation, and other factors leading to the progression of coronary atherosclerosis (8). Fifth, the analysis of APC by CCTA was not repeated during the follow-up. Therefore, we could not account for the potentially dynamic nature of HRPC in NCLs, which may provide incremental prognostic value (45). Sixth, imaging of APCs in combination with biomarkers, e.g., lipoprotein(a) and hs-CRP, or biomechanical parameters, such as perivascular fat attenuation and shear stress patients throughout the whole coronary vascular tree, holds promise for improving predictive power at the patient level (29, 46–48). The link between initiation or intensification of prevention with these imaging parameters on CCTA seems to be vital to improve the outcome of patients; however, there is no uniform agreement on use in evidence-based clinical decision-making (46). More studies are needed to further investigate the clinical impact and management adherence based on analysis of NCL by CCTA (38, 45). Seventh, it seems time-consuming to evaluate the quantitative analysis of APC. Recently, a multicenter and international study developed a deep learning-enabled system for rapid and automated APC quantification from CCTA, which can dramatically reduce analysis time at no expense of analysis accuracy (49). Finally, long-term medication status, which may have a significant impact on clinical outcomes, was not recorded (39).

## Conclusions

This is the first CCTA-based study investigating the natural history of NCLs in patients with DM and CCS after successful PCI for CL. During the 4-year follow-up, recurrent MACEs continued to occur and nearly half of these events were associated with NCLs. The use of CCTA can non-invasively detect ASPCs, such as a large plaque burden and LDNCP volume, a small MLA, or some combinations of these characteristics, which were predictive of MACEs arising from untreated NCLs. These results provided immediate prognostic value and may be considered a tool to guide the management of patients with DM and CCS.

## Data Availability

The raw data supporting the conclusions of this article will be made available by the authors, without undue reservation.
