# Supplementary material for: Identification of vulnerable non-culprit lesions by coronary computed tomography angiography in patients with chronic coronary syndrome and diabetes mellitus
Source: Front Cardiovasc Med. 2023 Mar 23;10:1143119. doi: 10.3389/fcvm.2023.1143119 (PMC10076802; doi:10.3389/fcvm.2023.1143119)
Supplement: Supplementary file 1 [file Presentation1.pdf]

Supplementary Material

Supplemental Methods

Baseline clinical data

Hypertension was defined as blood pressure of ≥140/90 mmHg or the use of anti-hypertension medication. Hyperlipidemia was defined as total cholesterol of ≥220 mg/dL, low-density lipoprotein cholesterol of ≥140 mg/dL, fasting triglycerides of ≥150 mm/dL or receiving treatment with oral lipid-lowering agents. Smoking was defined as current smoking or smoking in past 6 months. A family history of premature CAD was defined as diagnosis of the disease in a male first-degree relative before 55 years of age or in a female first-degree relative before 65 years of age. Renal insufficiency was defined as estimated glomerular filtration rate less than 80 mL/min/1.73 m^2.

Imaging protocols and analysis

All CCTA scans were performed with a second-generation dual-source CCTA scanner (Somatom Definition Flash, Siemens Medical Solutions, Forchheim, Germany). Sublingual nitroglycerine and heart-rate control by betablocker for a target heart rate ≤70 beats/min were administered as appropriate. A non-contrast-enhanced cardiac CT scan was acquired to using standard techniques[1]. Afterwards, A contrast enhanced CCTA was performed with detector collimation of 2×128×0.6 mm, slice thickness of 0.6 mm, gantry rotation time of 280 ms, heart rate adaptive pitch of 0.2–0.5, tube current of 290 to 560 mAs/rotation and tube voltage of 80–120 kV. Contrast volume was 60–90 mL followed by a normal saline of 50 mL and was injected intravenously in an antecubital vein. Bolus tracking is used to synchronize the arrival of contrast in the coronary arteries and the initiation of the scan, and the region of interest (ROI) was set at the root of the ascending aorta. Data acquisition was initiated with a delay of 5 seconds after signal attenuation threshold (100 HU) was reached in ROI. Image scan was triggered from 30 % to 80 % of the R-R interval.

The CCTA images were analyzed at a core laboratory (Tianjin Chest Hospital, Tianjin, China) by experienced radiologists and cardiologists who were blinded to the clinical and other imaging data using semiautomated software (Autoplaque, version 2.5; Cedars-Sinai Medical Center, Los Angeles, Calif) as previously described[2-4]. All segments ⩾2 mm in diameter were analyzed and the maximal degree of coronary area stenosis was defined as <30%, 30-50% and >50% according to the CAD-RADS(TM) Coronary Artery Disease – Reporting and Data System[5]. All lesion with area stenosis ⩾30% were identified in the present study.

Plaque components were quantified within the manually designated area using adaptive algorithms and automatically generated thresholds of calcified and non-calcified plaque (NCP). Low-density NCP (LDNCP) was defined as the portion of NCP with density levels ⩽30 Hounsfield units. Manual adjustments were made if necessary. The quantitative analysis of lesions included area stenosis, absolute volumes of NCP, LDNCP and total plaque, remodeling index and lesion length. Plaque burden was defined as the plaque volume normalized to the vessel volume (plaque volume×

100%/vessel volume), expressed separately for each plaque component. Qualitative analysis of APCs was performed according to the definitions from previous studies[2-4, 6-8] and established guideline[9], including area stenosis >50%, MLA <4mm^2, plaque burden >70%, LDNCP volume >30mm^3, positive remodeling (remodeling index>1.1), spotty calcification (diameter <3 mm in any direction, length of the calcium <1.5×vessel diameter, and width of the calcification <two-thirds of the vessel diameter) and NRS (ring-like attenuation pattern with peripheral high attenuation tissue surrounding a central lower attenuation portion).

ICA was attempted through the radial or femoral artery. The angiographic images were acquired with a GE INOVA-2000 single-plane system at a cine rate of 30 frames/s. A minimum of 8 projections were obtained (minimum of 5 views for the left coronary artery system and minimum of 3 views for the right coronary artery system). Each coronary segment with a >1.5mm diameter was analyzed for the presence of coronary diameter stenosis by 3 experienced cardiologist who were unaware of clinical data and CCTA results. The severity of angiographic disease was also assessed by SYNTAX score.

Follow-up and study endpoints

Contact information including telephone number, e-mail address and home address were collected before CCTA. All patients were followed up until January 2022. Cardiac death was defined as any death caused by cardiac disease or for which no other cause could be found. Myocardial infarction was defined according to the Fourth Universal Definition of Myocardial Infarction[10].

References

1. Hecht HS, Cronin P, Blaha MJ, Budoff MJ, Kazerooni EA, Narula J, Yankelevitz D, Abbara S. 2016 SCCT/STR guidelines for coronary artery calcium scoring of noncontrast noncardiac chest CT scans: A report of the Society of Cardiovascular Computed Tomography and Society of Thoracic Radiology. J Cardiovasc Comput Tomogr. 2017;11:74-84.

2. Gaur S, Øvrehus KA, Dey D, Leipsic J, Bøtker HE, Jensen JM, Narula J, Ahmadi A, Achenbach S, Ko BS, Christiansen EH, Kaltoft AK, Berman DS, Bezerra H, Lassen JF, Nørgaard BL. Coronary plaque quantification and fractional flow reserve by coronary computed tomography angiography identify ischaemia-causing lesions. Eur Heart J. 2016;37:1220-7.

3. Dey D, Schepis T, Marwan M, Slomka PJ, Berman DS, Achenbach S. Automated three-dimensional quantification of noncalcified coronary plaque from coronary CT angiography: comparison with intravascular US. Radiology. 2010;257:516-22.

4. Lee JM, Choi KH, Koo BK, Park J, Kim J, Hwang D, Rhee TM, Kim HY, Jung HW, Kim KJ, Yoshiaki K, Shin ES, Doh JH, Chang HJ, Cho YK, Yoon HJ, Nam CW, Hur SH, Wang J, Chen S, Kuramitsu S, Tanaka N, Matsuo H, Akasaka T. Prognostic Implications of Plaque Characteristics and Stenosis Severity in Patients With Coronary Artery Disease. J Am Coll Cardiol. 2019;73:2413-24.

5. Cury RC, Abbara S, Achenbach S, Agatston A, Berman DS, Budoff MJ, Dill KE, Jacobs JE, Maroules CD, Rubin GD, Rybicki FJ, Schoepf UJ, Shaw LJ, Stillman AE, White CS, Woodard PK, Leipsic JA. CAD-RADS(TM) Coronary Artery

Disease - Reporting and Data System. An expert consensus document of the Society of Cardiovascular Computed Tomography (SCCT), the American College of Radiology (ACR) and the North American Society for Cardiovascular Imaging (NASCI). Endorsed by the American College of Cardiology. J Cardiovasc Comput Tomogr 2016;10:269-81.
6. Williams MC, Kwiecinski J, Doris M, McElhinney P, D'Souza MS, Cadet S, Adamson PD, Moss AJ, Alam S, Hunter A, Shah ASV, Mills NL, Pawade T, Wang C, Weir McCall J, Bonnici-Mallia M, Murrills C, Roditi G, van Beek EJR, Shaw LJ, Nicol ED, Berman DS, Slomka PJ, Newby DE, Dweck MR, Dey D. Low-Attenuation Noncalcified Plaque on Coronary Computed Tomography Angiography Predicts Myocardial Infarction: Results From the Multicenter SCOT-HEART Trial (Scottish Computed Tomography of the HEART). Circulation 2020;141:1452-62.
7. Chang HJ, Lin FY, Lee SE, Andreini D, Bax J, Cademartiri F, Chinnaiyan K, Chow BJW, Conte E, Cury RC, Feuchtner G, Hadamitzky M, Kim YJ, Leipsic J, Maffei E, Marques H, Plank F, Pontone G, Raff GL, van Rosendael AR, Villines TC, Weirich HG, Al'Aref SJ, Baskaran L, Cho I, Danad I, Han D, Heo R, Lee JH, Rivzi A, Stuijfzand WJ, Gransar H, Lu Y, Sung JM, Park HB, Berman DS, Budoff MJ, Samady H, Shaw LJ, Stone PH, Virmani R, Narula J, Min JK. Coronary Atherosclerotic Precursors of Acute Coronary Syndromes. J Am Coll Cardiol 2018;71:2511-22.
8. Nerlekar N, Ha FJ, Cheshire C, Rashid H, Cameron JD, Wong DT, Seneviratne S, Brown AJ. Computed Tomographic Coronary Angiography-Derived Plaque Characteristics Predict Major Adverse Cardiovascular Events: A Systematic Review and Meta-Analysis. Circ Cardiovasc Imaging 2018;11:e006973.
9. Shaw LJ, Blankstein R, Bax JJ, Ferencik M, Bittencourt MS, Min JK, Berman DS, Leipsic J, Villines TC, Dey D, Al'Aref S, Williams MC, Lin F, Baskaran L, Litt H, Litmanovich D, Cury R, Gianni U, van den Hoogen I, A RvR, Budoff M, Chang HJ, H EH, Feuchtner G, Ahmadi A, Ghoshajra BB, Newby D, Chandrashekhar YS, Narula J. Society of Cardiovascular Computed Tomography / North American Society of Cardiovascular Imaging - Expert Consensus Document on Coronary CT Imaging of Atherosclerotic Plaque. J Cardiovasc Comput Tomogr 2021;15:93-109.
10. Thygesen K, Alpert JS, Jaffe AS, Chaitman BR, Bax JJ, Morrow DA, White HD. Fourth Universal Definition of Myocardial Infarction (2018). J Am Coll Cardiol 2018;72:2231-64.

CCTA-measured APCs	MACE (n=119)	No MACE (n=1129)	hazard ratio (95% CI)
	Number (%)		
Plaque burden >70%	270 (24)	139 (12)	4.02 (2.81-5.93)
LDNCP volume >30mm^3	84 (71)	447 (40)	2.99 (1.98-4.71)
MLA <4mm^2	78 (66)	491 (43)	2.07 (1.19-3.26)
LDNCP volume >30mm^3 + MLA <4mm^2	37 (31)	141 (12)	3.03 (2.13-4.32)
Plaque burden >70% + MLA <4mm^2	35 (29)	62 (5)	5.44 (3.89-7.71)
LDNCP volume >30mm^3 + Plaque burden >70%	50 (42)	88 (8)	6.86 (4.81-10.29)
LDNCP volume >30mm^3 + Plaque burden >70% + MLA <4mm^2	33 (28)	23 (2)	8.14 (5.92-13.30)

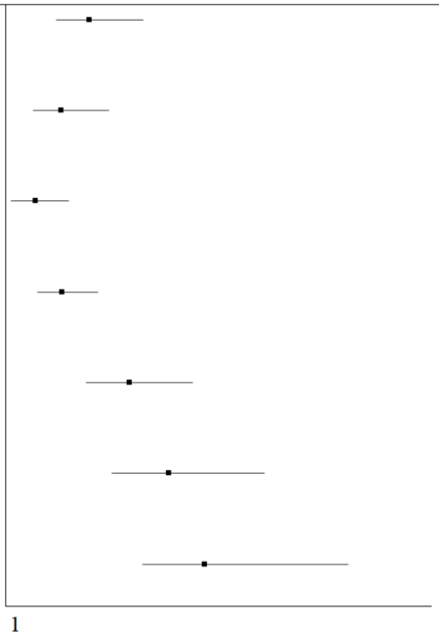

Supplemental Figure legends. Figure S1 Association between the study endpoints (regarding all indeterminate MACEs as NCL-related MACEs) and APCs of NCL

CCTA: coronary computed tomographic angiography; NCL: non-culprit lesion; MLA: minimum lumen area; LDNCP: low-density noncalcified plaque; APC: Adverse plaque characteristic; MACE: major adverse cardiovascular event; CI: confidence interval.
